# Supplementary figures and images for: Secondary Metabolism Gene Clusters Exhibit Increasingly Dynamic and Differential Expression during Asexual Growth, Conidiation, and Sexual Development in Neurospora crassa
Source: mSystems. 2022 May 31;7(3):e00232-22. doi: 10.1128/msystems.00232-22 (PMC9239088; doi:10.1128/msystems.00232-22)

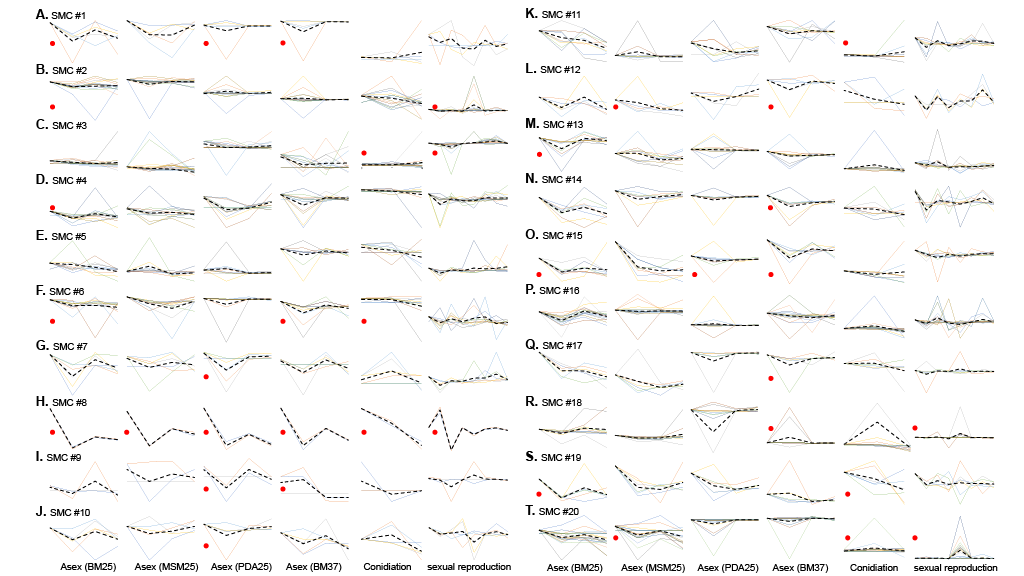

Supplement: FIG S1 [file msystems.00232-22-s0001.tif]
